# Fc receptors act as innate immune receptors during infection?

**DOI:** 10.3389/fimmu.2023.1188497

**Published:** 2023-07-26

**Authors:** Chaimaa Laassili, Fatiha Ben El Hend, Riad Benzidane, Loubna Oumeslakht, Abdel-Ilah Aziz, Rachid El Fatimy, Armand Bensussan, Sanae Ben Mkaddem

**Affiliations:** ^1^ Faculty of Medical Sciences, Mohammed VI Polytechnic University, Benguerir, Morocco; ^2^ INSERM U976, Université de Paris, Hôpital Saint Louis, Paris, France; ^3^ Institut Jean Godinot, Centre de Lutte Contre le Cancer, Reims, France

**Keywords:** FcR, innate immunity, bacterial clearance, ITAM bearing receptors, infection

## Abstract

Innate immunity constitutes the first nonspecific immunological line of defense against infection. In this response, a variety of mechanisms are activated: the complement system, phagocytosis, and the inflammatory response. Then, adaptive immunity is activated. Major opsonization mediators during infections are immunoglobulins (Igs), the function of which is mediated through Fc receptors (FcRs). However, in addition to their role in adaptive immunity, FcRs have been shown to play a role in innate immunity by interacting directly with bacteria in the absence of their natural ligands (Igs). Additionally, it has been hypothesized that during the early phase of bacterial infection, FcRs play a protective role via innate immune functions mediated through direct recognition of bacteria, and as the infection progresses to later phases, FcRs exhibit their established function as receptors in adaptive immunity. This review provides detailed insight into the potential role of FcRs as innate immune mediators of the host defense against bacterial infection independent of opsonins.

## Introduction

The human body is protected through a set of core mechanisms underlying interrelated innate and adaptive immune responses. Notably, Fc receptors (FcRs) expressed on innate immune cells bind mediators of adaptive immunity (immunoglobulins (Ig)/antibodies (Ab)). The crosslinking of FcR results in key biological and protective functions, including phagocytosis, antibody-dependent cellular cytotoxicity, and respiratory burst. These membrane-bound/soluble FcRs constitute a connection between humoral immunity and cellular effectors (1). Generally, FcRs are composed of an extracellular Ig-binding domain, a transmembrane domain, and an intracellular domain critical to signal transduction. Based on the signaling adaptors that are associated with FcRs (FcRγ/FcRβ), the signaling pathways activated differ (2). FcRs can even bear an ITAM (immunoreceptor tyrosine-based activation motif), ITAMi (inhibitory ITAM) (3) or ITIM (immunoreceptor tyrosine-based inhibitory motif) motifs. The different signaling pathways triggered by these motifs are crucial for whole-system homeostasis (3) and depend on the ligand and the receptor isoform (3, 4).

Five types of FcRs have been reported based on the Ig type: FcαRI binds IgA, FcδR binds IgD, FcϵR binds IgE, FcγR binds IgG, and FcμR binds IgM, and each type exhibits a specific physiological function and is activated under specific conditions (4). The FcαRI and FcγR, receptors belonging to the immunoglobulin superfamily that are highly expressed on monocytes, neutrophils, and dendritic cells (5). The interaction between these receptors and their specific immunoglobulins (mainly IgA or IgG, respectively) mediates responses against pathogens (opsonization) (6). IgA and IgG are the most prominent secretory immunoglobulins and play critical roles in immunity-based protection against pathogen invasion. This review provides insight into FcRs’ function and emphasis their potential role as innate immune receptors of bacteria and mediators of host defense during early stages of infection.

## FcαRI (CD89) function

IgA, a prominent serum immunoglobulin and the primary antibody class in mucosal secretions, plays a critical role in immune defense. It allows pathogen neutralization (7), complement activation (8), and interaction with host receptors, including the transferrin receptor (9–12) and FcαRI (13). In fact, the body expends significant energy in the production of IgA, implying that the advantageous immune protection conferred by IgA must be substantial. IgA is the second most common antibody in serum, second to IgG, with concentrations of approximately 2-3 mg/ml (14). Although serum IgA is largely monomeric, secretory IgA is predominantly polymeric, comprising mostly dimeric forms of two IgA monomers linked by one molecule in the J chains (15).

The IgA monomeric structural unit, like other that of immunoglobulins, consists of two identical heavy chains and two identical light chains grouped into two Fab regions and an Fc region connected by a flexible hinge region. Antigen recognition is achieved through by the action of the paired variable areas at the ends of the Fab arms, and the Fc region facilitates interactions with different receptors and effector molecules (16). In humans, there are two subclasses of IgA, IgA1 and IgA2, each encoded by a distinct gene. Several sequences in their heavy chain constant region vary (17). Surprisingly, whereas two IgA subclasses have been identified in humans, only one subclass has been found in rats (6). To date, five types of IgA receptors have been identified. FcαRI, the polymeric Ig receptor (PIgR), Fcα/μR, the transferrin receptor, and the asialoglycoprotein receptor (6).

The interaction between FcαRI and IgA complexed with antigens, can trigger a variety of cellular reactions, including phagocytosis, antibody-dependent cell cytotoxicity, and the release of inflammatory mediators and reactive oxygen species (ROS) (6). Importantly, IgA binding to FcαRI, results in the activation of intracellular signaling pathways, leading to the suppression of proinflammatory cytokine production and the promotion of anti-inflammatory cytokine production. This outcome ultimately leads to a reduction in the inflammatory response and contributes to tissue homeostasis and prevention of excessive inflammatory responses. FcαRI belongs to the immunoglobulin superfamily and carries a 206 amino acid extracellular region, a 19 amino acid transmembrane domain, and a 41 amino acid cytoplasmic region (18). The FcαRI extracellular region is composed of two Ig-like domains, EC1 and EC2, as well as six potential N-glycosylation sites (19). The signaling and cellular responses generated after FcαRI binding with IgA changes depending on the condition of the IgA molecules. The crosslinking of FcαRI by an immune complex containing IgA results in Src family kinase activation (20), and the tyrosine residues in FcRγ chain ITAM (ITAM consist of two tyrosine-containing boxes *(Tyr-X-X-Leu)* separated by seven amino acids) are phosphorylated by Lyn and Fyn (20). Phosphorylated ITAM tyrosine residues are docking sites for the tyrosine kinase Syk and trigger PI3K and PLCγ activating signaling pathways and calcium release (6, 21). The subsequent signaling cascades cause the aforementioned proinflammatory responses. In parallel, Src family kinases, after sequential phosphorylation, activate the Raf-1–MEK–MAP pathway, inducing a cascade of nuclear processes, including gene expression and the activation of transcription factors (6, 21, 22).

## Fcγ receptor’s function

IgG (immunoglobulin G) is one of the most prevalent proteins in human serum, accounting for around 10-20% of plasma protein. It is the most important of the five types of immunoglobulins in humans (23). IgG is composed of two identical heavy chains and two identical light (L) chains connected by inter-chain disulfide bonds. Additionally, IgG may be further subdivided into four subclasses in decreasing order of abundance: IgG1, IgG2, IgG3, and IgG4. Although they are more than 90% similar in amino acid sequence, each subclass has a distinct profile in terms of antigen binding, immune complex formation, complement activation, effector cell triggering, half-life, and placental transit (24).

FcγRs interact with the Fc portion of IgG and serve as a link between humoral and cell-mediated immune responses. Furthermore, studies have revealed that FcγRs function as receptors for innate immune opsonins (pentraxins) and serve as a link between innate and adaptive immunity (1). The classic FcγR family in humans is divided into three receptor subgroups based on structural homology, affinity differences, and selectivity for IgG subclasses (FcγRI (CD64), FcγRII (CD32), and FcγRIII (CD16)) (25). They can activate or inhibit immune functions such as phagocytosis, cytotoxicity, degranulation, antigen presentation, and cytokine production via immune tyrosine activating (ITAM) or inhibitory motifs (ITIM). The FcRs bearing ITAM motif include FcγRI, FcγRIIA/C and FcγRIIII). However the only one FcR bearing ITIM motif is FcγRIIB (1, 25).

Upon cross-linking of the activating FcγRs by immune complexes, different signaling pathways can be induced through ITAM, which normally exerts a sequential activation of protein tyrosine kinases of the Src-family followed by activation of the Syk tyrosine kinase (26, 27). This causes tyrosine phosphorylation of the ITAM, which leads to the recruitment of different downstream targets such as other kinases, adaptor molecules, and other signaling intermediates (28). Activation of PI3K results in the recruitment of phospholipase C (PLC) in numerous cell types (i.e. macrophages, mastocytes, etc.) which in turn generates a PLC mediated calcium release, triggering various effector functions (29, 30). Activating FcRs mediate cell functions such as phagocytosis, respiratory burst, and cytokine production (TNF-, IL-6), in antigen presenting cells such as macrophages and dendritic cells; in neutrophils and NK cells, they trigger antibody-dependent cellular cytotoxicity (ADCC) and degranulation; and in mast cells, they trigger degranulation (31, 32). As the only Fc receptor containing an ITIM, FcγRIIb suppresses IC-mediated cell activation, and when co-cross-linked with surface Ig, FcγRIIb suppresses B cell activation. When FcγRIIb interacts with other activating receptors, the ITIM is phosphorylated by a Src family kinase like Lyn, resulting in the recruitment of Src homology 2 domain-containing protein tyrosine phosphatase (SHP-1), SHP-2, and/or SH2-domain-containing inositol polyphosphate 5′ phosphatase (SHIP). SHIP can inhibit activation pathways mediated by pleckstrin homology-domain-containing kinases like PLC and Bruton’s tyrosine kinase (BTK), suppressing downstream events like Ig-induced calcium flux and decreasing multiple cellular functions induced by ITAM-containing receptors (33).

## FcR’s dual functions

**Figure 1:**
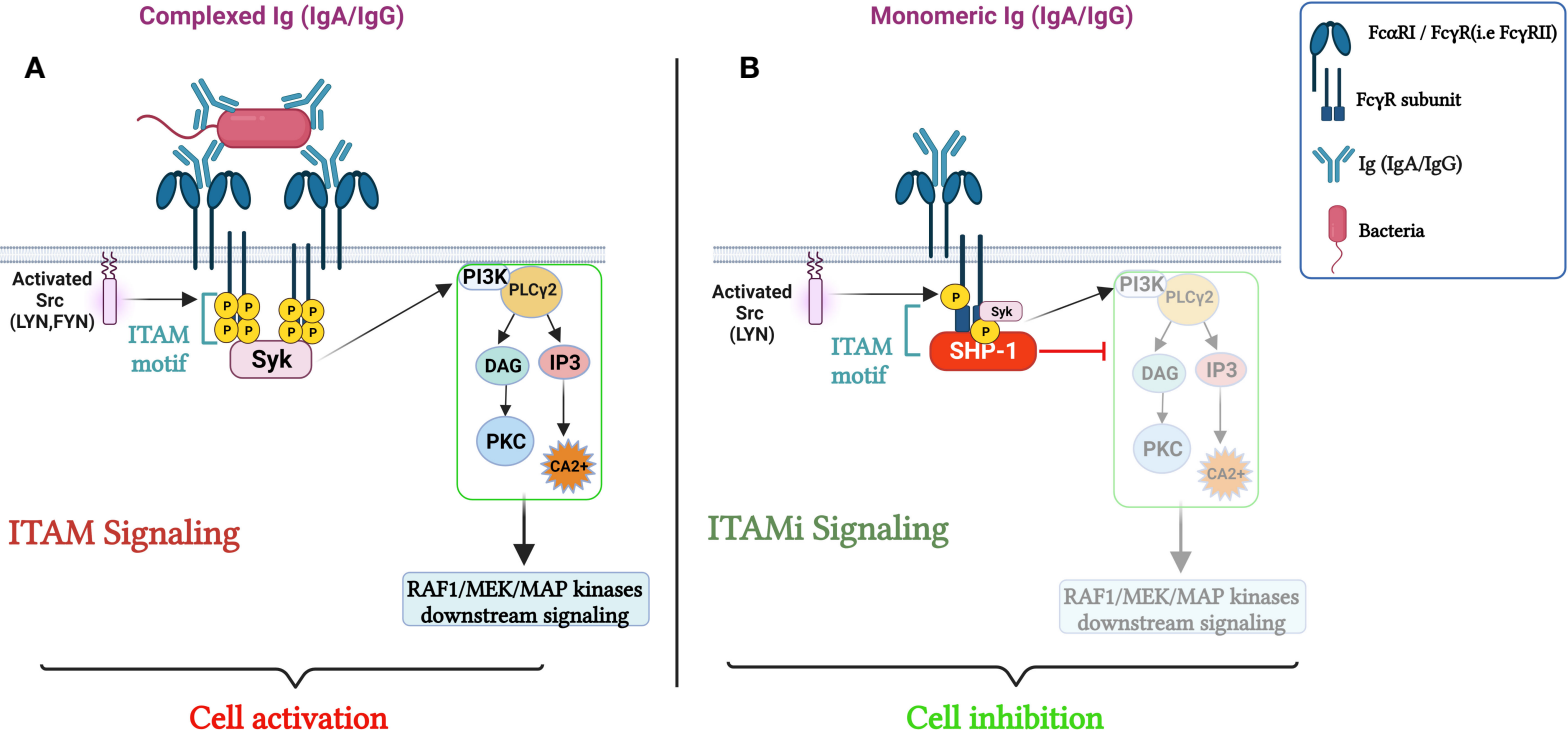
Role of FcRs (FcαRI/FcγRs) in the Regulation of cellular immune responses. **(A)** cell activation through FcRs mediated ITAM signaling. The crosslinking of the FcαRI by an immune complex containing IgA results in Src family kinases activation (Lyn and Fyn), followed by ITAM tyrosine residues phosphorylation, that will then serve as docking sites for the tyrosine kinase Syk, triggering PI3K, PLCγ activation signaling pathways and calcium release initiating pro-inflammatory responses. In parallel, sequential phosphorylation of Src family kinases, leads to the activation of Raf-1–MEK–MAP, launching a cascade of nuclear processes including gene expression and the activation of transcription factors. **(B)** ITAMi induced inhibition and homeostatic control. Monovalent targeting of the FcRs activates the Src kinase, Lyn, which partially phosphorylates the tyrosine within the FcRγ chain. The phosphorylated tyrosine serves for the transient recruitment of Syk followed by the stable recruitment of phosphatase-1 (SHP-1) to the FcRγ-chain. SHP-1 coordinates the anti-inflammatory response by dephosphorylating different proteins. Adapted from (21).

The FcR bearing ITAM motif (FcαRI, FcγRIII and FcγRIIA) can function as an inhibitory receptor via its ITAM motifs after FcR-monomeric immunoglobulins (IgA and IgG) interaction. The ITAM-mediated inhibitory signal is named ITAMi (34–36). This dual function of FcR plays an essential role in maintaining homeostasis (34–36). Monovalent or divalent targeting of FcR activates the Src kinase Lyn, which partially phosphorylates the tyrosine within ITAM. The phosphorylated tyrosine serves as a docking site for a tyrosine phosphatase-1 (SHP-1). This phosphatase inhibits the phosphorylation of different proteins induced by heterologous receptors, such as Toll-like receptors, involved in the pro-inflammatory responses. In the absence of pathogens, FcR-mediated ITAMi signaling prevents over-activation of the immune system and related tissue damage (3) (**Figure 1**). It has been discovered that ITAMi signaling, initiated by the binding of monomeric ligands to FcRs, resulted in its dynamic clustering with heterologous activating receptors, signaling effectors, and the inhibitory phosphatase SHP-1 within polarized intracellular clusters (36). These clusters are referred to as inhibisomes whose formation is always preceded by the recruitment of FcαRI and SHP-1 into lipid rafts (36). Moreover, Immunoreceptors convey signals based on their phosphorylation state, which is regulated by the protein kinases Lyn and Fyn that have opposing effects on ITAM receptors. This dual function has been demonstrated for FcαRI, FcγRIIA and FcγRIII and is exploited to develop therapeutic approaches for autoimmune and inflammatory diseases. Anti- FcαRI Fab was shown to induce ITAMi configuration by recruiting SHP-1 resulting in reduced rheumatoid arthritis (37). Furthermore, shifting FcγRIIA mediating ITAMi signaling was reported to potentially ameliorate inflammation (20). In the same line, intravenous Ig interaction with FcγRIIIA induced ITAMi signaling, thus preventing the development of autoimmune and inflammatory diseases (38). Interestingly, it was shown that FcγRIIIA is able to induce ITAMi signaling during infection favoring sepsis (39).

## CRP plays a key role in bacterial clearance through FcRs

In humans, the C- reactive protein CRP is a protein with expression that increase more than 1,000-fold in acute-phase responses associated with a severe inflammatory state, trauma and infection (40). CRP is composed of noncovalently bound subunits comprising 206 amino acid residues with a molecular weight of ~23 kDa. One face of the pentamer carries five sites of calcium-dependent substrate binding (41). When bound to its receptor, CRP can induce a Quellung-like reaction with some pneumococcal serotypes (42) and has been demonstrated to enhance the phagocytosis of bacteria upon binding to it (43). The crucial role of CRP in pathogen clearance has been established. Kaplan and Volanakis showed that CRP in reaction with pneumococcal C-polysaccharide 3 induces the consumption of the human complement components and the conversion of C3 (44). Moreover, Siegele and colleagues demonstrated that CRP activates the classical complement pathway (44, 45) the administration of human CRP has been found to be protective against pneumococcal infection in mouse models, highlighting the role of CRP in reducing bacteremia and promoting survival in infected murine models (46). Interestingly, it has been demonstrated that the aggregate IgG inhibited the CRP-dependent phagocytosis (47) indicating a potential direct link between CRP and FcγRs. Indeed, using FcγRI and FcγRIIA transfected COS cells, the interaction between CRP and FcγRs was established, reporting that the FcγRIIA is a primary receptor for CRP on human and mouse macrophages/monocytes and, neutrophiles. The protective role of CRP against pneumococcal infection was proven. However, this protective function was independent of FcγRs. Nevertheless, CRP protection was completely dependent on FcγRs in a mouse model during endotoxin shock (48). Moreover, Lu and colleagues demonstrated that CRP could bind FcαRI resulting in ERK phosphorylation, degranulation, and cytokine production in FcαRI-transfected cells. Furthermore, CRP induces FcαRI expression, bacterial phagocytosis, and TNF-alpha release in neutrophils (49).

Taken together, CRP interacts with FcRs mediating cell activation and bacterial clearance. However, prior to the increase of CRP and pathogen specific Igs production, innate immunity plays an important role in fighting against pathogens. One prominent mechanism of the innate immune response is the direct host recognition of the innate immune receptor on phagocytes and pathogens. In addition to the classic interaction between FcRs and their specific immunoglobulins, FcRs could interact directly with bacteria during infection.

## Direct FcαRI – bacteria interaction protects against sepsis

**Figure 2:**
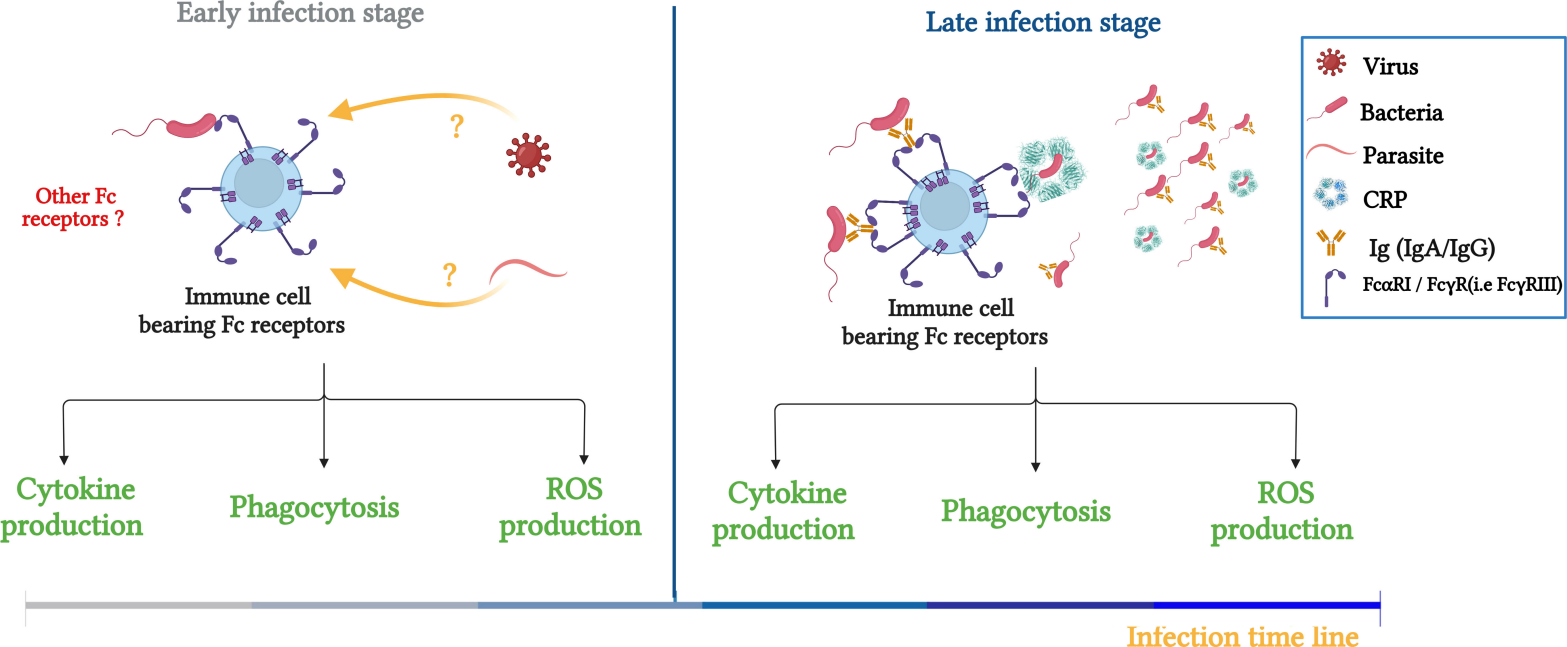
Schematic illustration summarizing the role of FcRs (FcαRI/FcγRs) in bacterial clearance and potential contribution to innate immunity responses during infection. Left panel: FcRs serve as an innate receptor during the early phase of infection by interacting directly with bacteria, the question about the FcRs to interact directly with other pathogens, such as viruses and bacteria is maintained. Right panel: During the late phase, FcRs are involved in innate and adaptive immune responses through double interaction with opsonized bacteria (IgA, IgG and CRP) and non-opsonized bacteria. Adapted from (50).

Recently, De Tymowski et al. investigated whether the ITAM-bearing FcR, FcαRI (6), can directly bind bacteria (in the absence of their natural ligands, such as IgA and CRP). Indeed, they showed that soluble recombinant FcαRI bound both gram+ and gram- bacteria (*E. coli* and *Streptococcus pneumonia* (*S.P*) respectively). This direct FcαRI–bacteria interaction was confirmed using bone marrow-derived CD14^+^ macrophages and CD11c^+^dendritic cells in the absence of IgA and CRP. Moreover, the FcαRI–bacteria interaction resulted in the production of cytokines, reactive oxygen species, and phagocytosis let to bacteria killing. These findings were confirmed with human blood monocytes and macrophages, suggesting a protective function for FcαRI against *S.P* and *E. Coli*) (50). Furthermore, the cell responses mediated through FcαRI–bacteria interactions protected mice against sepsis via the induction of a transient and efficient inflammatory response and an increase in bacterial clearance and survival that depended on the FcRγ subunit. A layer of functional complexity is added to the IgA–FcαRI interaction, which plays a crucial role in host defense and both activating and inhibitory responses. Additionally, because no IgA antibodies specific for the bacterium specific were found before 48 hours of infection, the importance of FcαRI in innate immunity prior to its activity in adaptive immune responses is evident. This study suggested that during the early phase of bacterial infection, FcαRI plays a protective role in innate immunity through its direct recognition of bacteria, and as the infection progresses, it exerts a conventional effects as a receptor in adaptive immunity mediated by binding IgA and CRP (**Figure 2**) (50).

Additionally, the same study has demonstrated that human serum IgA does not completely block bacterial binding to FcαRI, indicating that a proportion of free FcαRI remains free to capture bacteria (50). Additionally, although CRP in the bloodstream may further reduce the likelihood that FcαRI remain unbound, the immune system is dynamic, showing the potential for inducing changes in the availability of IgA and CRP in response to different physiological conditions or pathological stimuli. Therefore, it is possible that, in certain circumstances, unbound FcαRI may play a more significant role in bacterial entrapment. This scenario is, indeed, represented by three cases: Langerhans cells (LCs) (51), newborns (52), and IgA deficiency (53). i) A study aiming to determine FcαRI expression on interstitial-type dendritic cells and Langerhans-type dendritic cells showed that FcαRI was expressed only on intestinal-type dendritic cells but not on Langerhans cells (*in situ* and *in vivo*) (51). In fact, this expression led to functional outcomes, the activation of IL-10 production. This means that interstitial-type dendritic cells may express FcαRI to sense subepithelial tissue integrity. On the other hand, TGF-b1 downregulates FcαRI expression on human epithelial Langerhans cells *in situ*, making it undetectable via immunohistochemically approaches, indicating that LCs may not engage IgA immune complexes inside the epithelium when the epithelial barrier is not disrupted (51). ii) Furthermore, a newborn’s immune system relies mainly on the IgG antibodies they have received through the maternal placenta, and these antibodies fight against pathogens by initiating phagocytosis and activating the complement system. However, in newborns, the inflammatory response is inoperant. Immune response equilibrium is reached through breastfeeding, which provides the neonate with large amounts of secretory IgA (SIgA), which is incapable of inducing inflammation. This defense mechanism is necessary for the newborn to prevent damage to his or her fragile mucosal tissues (52). However, a critical question remains: How do neonates who are not breast fed defend themselves against pathogens? Is their defense based solely on IgG antibodies, which can be energy intensive and result in symptoms such as pain, loss of appetite, and fever, potentially impacting the neonate’s growth and development? Furthermore, what is the significance of FcαRI expression prior to the production of IgA, as it has been reported that FcαRI expression is independent of its ligand? The observation of newborns leads to speculation about the role of FcαRI in innate immunity. iii) Similar to the previous scenario with newborns, is selective IgA deficiency, described as a low IgA level, where IgM and IgG levels are unchanged (54). No one can deny the susceptibility of IgA-deficient patients to infections. However, this susceptibility is only true for one-half of patients; other half of patients do not develop recurrent infections. To date, no explanation has been given for this difference (54), taking into consideration that IgA-deficient patients still express FcαRI (53). The previously mentioned reinforces the idea that FcαRI expression is independent of its natural ligand and points to a possible direct interaction of FcαRI with pathogens in absence of IgA.

## Direct FcγRs– pathogen interaction role during infection

Sepsis during infection, a significant cause of mortality globally, is caused by proinflammatory processes and deficient bacterial clearance. By clearing pathogens through host innate receptors, phagocytic cells play an important role in sepsis prophylaxis. In fact, host interaction with opsonized bacteria via the FcγRIII, an IgG receptor was established to induce phagocytosis. However, it has been shown that FcγRIII, directly binds *E. coli* (gram-negative bacilli) independent of opsonins (IgG and CRP). This direct interaction induces activation of an ITAMi signaling pathway, which is characterized by the activation and recruitment of SHP-1 to FcγRIIIA. SHP-1, a phosphatase, dephosphorylates PI3K, decreasing the rate of *E. coli* phagocytosis and increasing the production of TNF-alpha. Using FcγRIIIA-KO mice, Da Silva et al, showed increased survival due to *E. coli* phagocytosis and decreased production of the proinflammatory cytokine TNF-alpha (39), suggesting a deleterious role for FcγRIIIA–bacteria interactions, through the FcRγ subunit, during infection by promoting sepsis. This study revealed another area of investigation into the ability of other FcRs, such as FcαRI, to interact directly with pathogens and the physiological implications of these interactions. As a continuation of the study of Silva and colleagues conducted exclusively with gram-negative bacteria (39), De Tymowski et al., investigated the potential interplay between *S.P* and FcγRIIIA. FcγRIIIA was found to play opposite roles in sepsis, depending on the type of bacteria involved (and the organ infected), and FcαRI expression was associated with a protective function triggered by the *S.P* and FcγRIIIA interaction, suggesting that FcαRI counteracts the deleterious effect of FcγRIIIA activation via the action of upstream pathways in the studied cecal ligation and puncture (CLP)-induced model (a commonly used procedure for modeling sepsis *in vivo*) (50).

Another potential case where FcγRs can interact directly with pathogens, is the central nervous system (CNS). The CNS is highly protected from pathogen infection due to IgA-secreting plasma cells adjacent to dural venous sinuses (55) and barriers such as the blood brain barrier (BBB). However, some pathogens (bacteria, viruses, fungi, etc.) enter the brain, causing deleterious infections and high mortality rates (56). These pathogens include enteroviruses, neurotropic viruses, Streptococci, etc (57). Microbial pathogens gain access to the brain by either crossing the BBB or the blood–cerebrospinal fluid barrier. Notably, three routes of entry are plausible: 1) Transcellular penetration occurs when bacteria [*E. coli, S. P* and others (58–61)] adhere to epithelial/endothelial cells via receptor-mediated mechanisms (56), 2) Paracellular entry as a result of the disruption of the tight junctions between brain barriers due to microbial toxicity of pathogens such as *E. coli* K1*, H. influenza* B *and S. P* (62), and 3) Trojan horse penetration, in which pathogens are internalized within host peripheral immune cells. This entry mechanism has been identified for *B. pseudomallei* (63), *S. suis* (64) and other pathogens. Additional entry routes involve olfactory/trigeminal nerves or nasal cavities (65). Nevertheless, the brain is endowed with cells that guarantee its integrity and protect it from these invasions; these protective cells are called microglia, the macrophages of the brain (66). They have been shown to express all classes of FcRs (67). The function of FcRs in the brain was clearly elucidated in the contexts of neurodegenerative (68) and immunological diseases (69), but what about microbial infections? In fact, incubation of microglia with IgG immune complexes resulted in phagocytosis, oxidative bursts and ADCC (70). Furthermore, FcRs expression on microglia was found to be decreased following treatment with TNF alpha, IFN gamma, IL-1 or IL-4 (71, 72). Nevertheless, those results were reported only *in vitro*. Among questions arising from these investigations: What is the significance of microglia expressing FcRs when considering that Igs cannot cross the BBB? One hypothesis suggests that microglia express FcRs to make direct contact with bacteria in the absence of opsonin. Moreover, recent studies have revealed that B cells can also be found in the nervous system, indicating their involvement in neurological processes. Important aspects of B cells in the nervous system include ab production: B cells in the CNS can produce antibodies locally, thus help in combating localized infections and maintaining immune homeostasis. However, before abs production, is there a direct interaction between bacteria and FcRs? Furthermore, the atypical FcγR, neonatal Fc receptors (FcRn), was suggested to interact directly with viruses. In fact, FcRn may transfer IgG from mother to fetus through the placenta, providing newborns with humoral immunity during pregnancy. Under normal physiological conditions, FcRn prolongs the half-life of IgG by preventing its catabolism, which plays a central role in antigen presentation and immune complex clearance (73, 74). However, a recent study highlighted the implication of FcRn in Zika virus (ZIKV) infection and proposed its association with higher susceptibility to infection since viral replication was higher in cells that overexpressed FcRn than in those that did not (73). In addition, decreased FcRn expression was associated with decreased RNA virus production. In the maternal placenta, ZIKV infection reduced the FcRn mRNA and protein levels. The possibility of a direct interaction of FcRn with the E protein of the glycoprotein envelope of a virus was suspected, explaining the deleterious role of FcRn (73). Interestingly, Xin. Z et al. have further highlighted the deleterious role of FcRn, noting that it bound directly to enterovirus B via its Fc gamma receptor and transporter (*FCGRT)*-encoded subunit, triggering the virus-uncoating process (75).

## IgA and bacteria glycosylation similarity

IgA and FcαRI are both glycosylated proteins. Glycosylation has been suggested to possibly affect the stability of IgA, but not its affinity for FcαRI. Moreover, it has also been reported that the glycosylation of FcαRI markedly affects the affinity of the IgA–FcαRI interaction (76). Furthermore, abnormal IgA glycosylation has been associated with several diseases, making it a promising biomarker for diagnosis, prognosis, and treatment (77). IgA nephropathy (IgAN) is a good example of ectopic glycosylation of IgA causing disease, as IgAN is characterized by deficient galactosylation of the glycoform IgA1(Gd-IgA1) hinge region, causing the release of proinflammatory mediators that lead to glomerulosclerosis, inflammation, and renal injury (78, 79). The same condition (deficient galactosylation of Gd-IgA1) plays a fundamental role in IgA vasculitis (80). In addition, abnormal glycosylation/galactosylation has been reported in autoimmune diseases, notably Crohn’s disease, systemic lupus erythematosus, and rheumatoid arthritis (77, 81). Taken together, these studies can lead one to deduce that the glycosylation/galactosylation state of N-glycans are major contributors to IgA–FcαRI interplay. The cell wall structure of both Gram+ and Gram- bacteria has been shown to be composed mainly of peptidoglycans forming a network of polysaccharide strands linked by means of peptide bridges (82, 83). One hypothesis based on an analogy of this N-glycan structure suggests that the bacteria interact with FcαRI in a manner similar to that of IgA with FcαRI.

## IgG like domains: potential bacterial motifs ruling FcγR-bacteria interaction

Bacterial infections can cause severe acute symptoms such as fulminant organ failure and high death rates. Thus, understanding host-pathogen interactions might reveal crucial information regarding novel immune evasion mechanisms. Fc receptors elicit a wide range of biological reactions, including both activating and inhibitory effects. Beppler and colleagues identify two new FcγRIIIa ligands with structural similarities to those discovered in the WzxE proteome: a translocase involved in the transbilayer movement of substrates involved in the assembly of cell surface polysaccharides that resembles IgG. In other words, E. coli (gram negative bacteria) contains an IgG like domains by which it interacts with the FcγRIIIa (84). In the same line, a study conducted on *Streptococcus agalactiae* (gram positive bacteria), the primary factor inducing neonatal pneumonia, sepsis, and meningitis. Gram-positive bacteria’s cell wall contains proteins and proteinaceous filaments, such as pili or fimbriae, that are covalently bonded to the cell wall for interactions with the host, resulting in commensal or deleterious connections. Genomic analysis of *Streptococcus agalactiae* reveals the crystal structure of the pilin GBS52, which encompasses two IgG-like fold domains, N1 and N2 that play a role in pathogen adherence (85). All the aforementioned suggests a potential role of the IgG like domains on bacterial cells walls to rule FcγR-bacteria interaction in the early phase of infection.

## Discussion

This review reports convincing core of evidence demonstrating the major role of ITAM-bearing FcRs (FcγR and FcαRI) (6) in innate immunity during infection. FcRs were shown to directly capture bacteria independently of their cognate ligands, and were suggested to play a role in the early phase of the infection before antibodies’ production leading to either inhibition or activation of key immune pathways. Nevertheless, it is important to delve further into the mechanisms underlying these interactions. For example, a study by Beppler and colleagues showed that FcγRIII interacted with *E. coli* through the bacterial WzxE motif (84), but the same motif was not found to play a role in the interaction between FcαRI and *E. coli* (50). Moreover, the role of N-linked glycans in the FcαRI–IgA interaction has been examined, and certain N-linked sugar moieties on FcαRI have been demonstrated to influence receptor binding to IgA (86, 87). Furthermore, gram-positive and gram-negative bacteria carry N-glycan groups on their membranes (88), suggesting that the interaction between bacteria and FcαRI may be mediated through polysaccharides (N-glycan groups) on the extracellular domains of both parties, which is an interesting line of inquiry. Furthermore, a recent study showed that the activation of Mincle, an FcRγ chain-coupled C-type lectin receptor expressed at a low level on myeloid cells, was triggered by *Leishmania major* (a eukaryotic parasite). In fact, *Leishmania major* activated a Mincle-dependent inhibitory axis involving the activation of SHP-1 and its coupling to the FcRγ subunit. This ITAM inhibitory signaling pathway was shown to dampen adaptive immunity to infection (89), suggesting another area of investigation related to determining whether the FcR**s** interactome is limited to bacteria only or extends to viruses and parasites and the consequences of these potential interaction (**Figure 2**); is this potential interaction protective (i.e., FcγRIIIA and *S.P*) or deleterious (FcγRIIIA and *E. coli*)? In the same line as the interaction of FcRs with nonbacterial pathogens and the consequences of their interplay, FcRn was shown to have a deleterious effect when interacts directly with viruses [Zika (73, 90) and cytomegalovirus (91)].

## Conclusion and outlook

The aforementioned solid core of evidence suggests a dual role of FcRs during the early phase of infection as innate immune receptors that can bind bacteria directly independently of opsonins and their natural ligands. This interaction can either exert a protective or a deleterious effect during sepsis. The spectrum of FcR-activated signaling pathways and the dual roles of FcRs as innate immunity receptors should be expanded. Interventions targeting of FcRs may not only decrease proinflammatory mediator production but may also enhance phagocytosis, anti-inflammatory mediator production, and sepsis attenuation more efficiently; moreover, a new role may be attributed to FcRs in innate immune responses in addition to their classical functions.

## Author contributions

CL: conceptualization, manuscript writing, figure artwork. FB: writing, review, editing. RB: review, editing. I-AA: review, editing. LO: review, editing. RE: editing. AB: review & editing, supervision, project administration. SB: conceptualization, writing -review & editing, supervision, project administration. All authors contributed to the article and approved the submitted version.
